# A Dew-Condensation Sensor Exploiting Local Variations in the Relative Refractive Index on the Dew-Friendly Surface of a Waveguide

**DOI:** 10.3390/s23052857

**Published:** 2023-03-06

**Authors:** Subin Hwa, Eun-Seon Sim, Jun-Hee Na, Ik-Hoon Jang, Jin-Hyuk Kwon, Min-Hoi Kim

**Affiliations:** 1 Department of Creative Convergence Engineering, Hanbat National University, Daejeon 34158, Republic of Korea; 2 Department of Electrical, Electronics & Communication Engineering Education, Chungnam National University, Daejeon 34134, Republic of Korea; 3 FarmAI Lab, Jinong, Anyang-si 14067, Republic of Korea; 4 Research Institute of Printed Electronics & 3D Printing, Industry University Cooperation Foundation, Hanbat National University, Daejeon 34158, Republic of Korea

**Keywords:** dew-condensation sensor, refractive index, dew-friendly surface, optical waveguide, water, specific heat

## Abstract

We propose a sensor technology for detecting dew condensation, which exploits a variation in the relative refractive index on the dew-friendly surface of an optical waveguide. The dew-condensation sensor is composed of a laser, waveguide, medium (i.e., filling material for the waveguide), and photodiode. The formation of dewdrops on the waveguide surface causes local increases in the relative refractive index accompanied by the transmission of the incident light rays, hence reducing the light intensity inside the waveguide. In particular, the dew-friendly surface of the waveguide is obtained by filling the interior of the waveguide with liquid H_2_O, i.e., water. A geometric design for the sensor was first carried out considering the curvature of the waveguide and the incident angles of the light rays. Moreover, the optical suitability of waveguide media with various absolute refractive indices, i.e., water, air, oil, and glass, were evaluated through simulation tests. In actual experiments, the sensor with the water-filled waveguide displayed a wider gap between the measured photocurrent levels under conditions with and without dew, than those with the air- and glass-filled waveguides, as a result of the relatively high specific heat of the water. The sensor with the water-filled waveguide exhibited excellent accuracy and repeatability as well.

## 1. Introduction

Precision agriculture with modern information technologies has recently attracted significant attention as a countermeasure to resolving food insecurity [1,2,3,4,5]. Gathering accurate information on the conditions of crops and the surrounding environment, such as weight/size, infection, biomass, humidity, and temperature, is one of the prerequisites for the implementation of precision agriculture [6,7,8,9]. Therefore, it is essential to develop appropriate sensor components that enable the effective collection of such information.

Water, which plays a vital role in the growth of crop plants, is the main constituent of the tissues of most creatures. However, water that fills the internal parts of the crops can be a fundamental cause for dew condensation on the surfaces of crops. Water is known to have a specific heat approximately four times higher than air at room conditions [10,11]. In a greenhouse where the inner air temperature rises rapidly in the early morning, the surfaces of water-containing crops maintain a relatively low temperature range. Consequently, dew condensation is facilitated by humid air when the surface temperature of crops is lower than the dew-point temperature [12,13,14,15,16].

Dew condensation has detrimental influences on the quality of crops, promoting fungal or bacterial diseases [14,15,16,17]; therefore, it is necessary to detect dew condensation in order to take remedial action. Various sensors for detecting dew condensation, including the resistive type, the time domain reflectometry (TDR) type, and the fiber optic type, have been developed [18,19,20,21,22,23,24,25,26,27,28]. Resistive types are low-cost sensors, but corrosion of the electrodes can negatively affect their repeatability. Although the TDR type can achieve a relatively high accuracy, it is relatively expensive. Fiber optic types are considered advantageous for implementing low-cost sensors and a good repeatability thereof. Moreover, it is widely known that fiber optic types can be relatively advantageous in terms of immunity to electromagnetic interference. However, without the help of certain precise thermometers and dew prediction algorithms, there are limitations in developing a dew-condensation sensor for specific surfaces, such as crops, through previous fiber-optic-type techniques. The use of additional thermometers for measuring the surface temperature of crops or ambient air is, in turn, likely to increase the entire cost of the sensor system. In addition, plasmonic, evanescent, and stack types can be employed for measuring dew condensation [29]; however, these types have a potential limitation to elevating sensing performance due to the difficulty of finding and modifying sensing layer materials suited to specific target analytes, such as dew. Hence, it is necessary to develop a low-cost high-performance dew-condensation sensor with high accuracy and excellent repeatability. Recently, a sensor device for detecting soil moisture, which exploits a moisture-induced variation in the relative refractive index on the outer surface of the air-filled waveguide, has been devised and reported [30]. It is possible to implement a dew-friendly surface for the waveguide by filling its interior with water, considering the high specific heat of the water. This implies that a new application for detecting dew condensation can be developed by transforming the soil-moisture sensor technology.

In this work, a sensor device, which detects dew condensation by exploiting a variation in the relative refractive index on the surface of the water-filled waveguide was developed. The waveguide geometry was designed to have the curvature for the total reflection of the incident light at the waveguide surface under conditions without dew. Variations in the internal optical paths of the waveguide were explored by inserting the absolute refractive indices of various filling materials (water, oil, air, and glass) for the waveguide through simulations. The dew-condensation sensors were fabricated with water, air, and glass as internal media for the waveguide and their detection accuracy was examined. Finally, the repeatability and uniformity of the dew-condensation sensor with the water-filled waveguide were examined.

## 2. Materials and Methods

**Figure 1:**
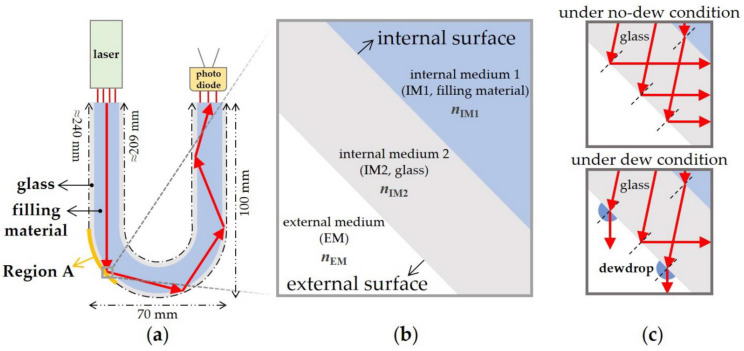
(**a**) Schematic diagram of the sensor, (**b**) key denotations for the constituent media and surfaces of the sensor as well as those for the absolute refractive indices of the media, and (**c**) the path difference between the incident light rays, with and without dew.

The dew-condensation sensor consists of a U-shaped glass waveguide for light propagation, a light-emitting part at one end of the waveguide, and a light-receiving part at the other end of the waveguide. The waveguide was filled with water, glass, or air as its internal medium. The air-filled waveguide is a hollow pyrex-glass fiber without an additional filling material, while the glass-filled waveguide is a solid pyrex-glass fiber that has no internal empty space. The water-filled waveguide was obtained by filling a hollow pyrex-glass fiber with deionized water. The waveguide core had a 7.0 mm diameter, the thickness of the cladding was 1.5 mm, and the length of the waveguide was ≈209 mm (inner) to ≈240 mm (outer) (Figure 1a). A laser (EPXFAVJH, AMYTA) with a wavelength of 650 nm was used for emitting light. A photodiode (S1223, HAMAMATSU) was used for receiving light. The sensor and a heater were installed in an acrylic box with a volume of 1 m × 1 m × 0.6 m. The evaporation of moisture from the soil in a greenhouse, due to solar radiation or ambient temperature rise, can increase the absolute humidity of the greenhouse air in the early morning. In our experiments, such an increase in absolute humidity was imitated by evaporating water from the heater in the acrylic box. Specifically, to induce dew condensation on the waveguide surface, water was evaporated from the heater at 200 °C. Driving circuits for the light-emitting part and the light-receiving part were connected to the microcontroller to construct the sensor system. In detail, the driving circuits consisted of a part for the signal conversion/amplification of the photodiode and one for the low-power operation of the laser. The laboratory conditions were 15% RH/17 °C.

For optical simulation, a commercial finite element method program (LightTools 9.0, Synopsys) was used. The light propagation through a waveguide was simulated with a single wavelength of 650 nm for the light. The light intensity at the end of the waveguide was calculated with and without simulated dew condensation on the waveguide surface, respectively. Various refractive indices were inserted for the internal medium of the waveguide.

## 3. Results and Discussion

### 3.1. The Basic Structure and Working Principle of the Dew-Condensation Sensor

As shown in Figure 1a, the waveguide of the sensor was designed to have the external surface, i.e., Region A, where the first total reflection of the incident light occurs under conditions without dew by designing a proper curvature for the waveguide and by filling the waveguide with a material whose refractive index is larger than the external medium, i.e., ambient air (Figure 1a). Figure 1b shows the key denotations for this study. Under conditions with dew, the transmission of the light at Region A is induced by a local variation (i.e., a local increase) in the relative refractive index at Region A (Figure 1c), since the absolute refractive index of a dewdrop, i.e., water, is higher than that of air. Consequently, the measured photocurrent at the light-receiving part decreases, hence providing information on dew condensation.

### 3.2. The Key Geometric Design for the Dew-Condensation Sensor

**Figure 2:**
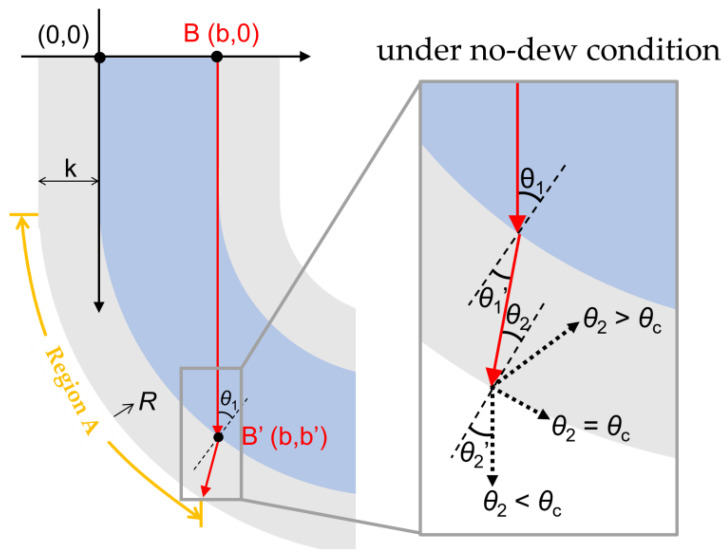
The geometry of the waveguide with curvature and the design strategy in which the condition of the total reflection at the external interface is considered.

It was necessary to design a proper curvature for the waveguide in order to prevent a waste of incident light due to the transmission at Region A under conditions without dew. If the curvature is excessively large, plenty of incident rays are transmitted at Region A regardless of dew formation. In addition, an excessively small curvature for the waveguide is undesirable in terms of device miniaturization and light weighting. Accordingly, the curvature was designed to make the incident ray satisfy $\theta_{2}>\theta_{c}$ at x=b without excessively reducing the curvature (Figure 2), where *θ*_2_ is the incident angle at the outer interface and *θ*_c_ the critical angle yielding total reflection under conditions without dew. Snell’s law for the external interface is written as $n_{\mathrm{IM}2}\sin\theta_{2}=n_{\mathrm{EM}}\sin\theta_{2}\prime$, where $n_{\mathrm{IM}2}$ is the absolute refractive index of the internal medium 2, $n_{\mathrm{EM}}$ is that of the external medium, and *θ*_2_’ the refraction angle. By inserting $\theta_{2}\prime =90\;{}^{\circ}$, the *θ*_c_ can be written as:

(1) $$\theta_{c}=\sin^{-1}\left(\frac{n_{\mathrm{EM}}}{n_{\mathrm{IM}2}}\right)$$

where *n*_EM_ is ≈1 representing the absolute refractive index of air and *n*_IM2_ 1.468 as that of glass. Snell’s law for the internal surface is written as $n_{\mathrm{IM}1}\sin\theta_{1}=n_{\mathrm{IM}2}\sin\theta_{1}\prime$, where *θ*_1_’ is ≈*θ*_2_ and $n_{\mathrm{IM}1}$ is the absolute refractive index of the internal medium 1. *θ*_2_ can then be expressed as:

(2) $$\theta_{2}=\sin^{-1}\left(\frac{n_{\mathrm{IM}1}}{n_{\mathrm{IM}2}}\;\sin\;\theta_{1}\right)$$

Considering Equations (1) and (2), $\sin\;\theta_{1}\;$ should be equal to $\frac{n_{\mathrm{EM}}}{n_{\mathrm{IM}1}}$ for $\theta_{2}=\theta_{c}$. In the geometry of the waveguide, $\sin\;\theta_{1}$ is equal to $\frac{R-k-b}{R-k}$ ($=1-\frac{b}{R-k}$) where *R* is the radius of curvature, *k* is the glass thickness of the waveguide (i.e., the thickness of the cladding), and *b* is the inner width of the waveguide (i.e., the diameter of the core) (Figure 2). Therefore, we can obtain the following condition for *R*, putting $\frac{n_{\mathrm{EM}}}{n_{\mathrm{IM}1}}=1-\frac{b}{R-k}$:

(3) $$R=\frac{b}{1-\frac{n_{\mathrm{EM}}}{n_{\mathrm{IM}1}}}+k$$

Based on Equation (3), *R* values calculated for water ($n_{\mathrm{IM}1}=1.330$), oil ($n_{\mathrm{IM}1}=1.405$), and glass ($n_{\mathrm{IM}1}=1.468$) were 29.7, 25.8, and 22.5 mm, respectively. For the actual design and fabrication, *R* of 35 mm, which is larger than all these calculated *R* values, was carefully chosen to render the incident ray at x=b satisfy $\theta_{2}>\theta_{c}$ for all the materials (water, oil, and glass) under conditions without dew.

### 3.3. Optical Simulation with Different Filling Materials for the Waveguide

**Figure 3:**
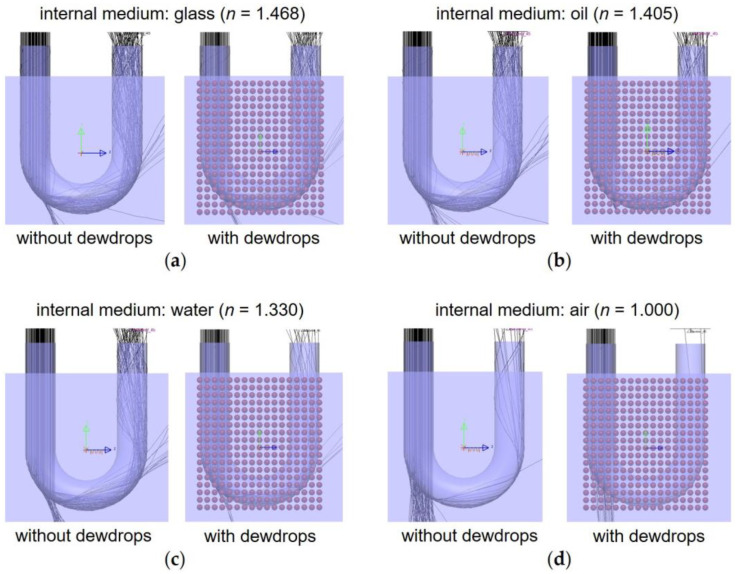
Optical simulation results (the light wavelength: 650 nm). Optical paths obtained with various internal media for the waveguide, i.e., (**a**) glass, (**b**) oil, (**c**) water, and (**d**) air.

The optical simulation was performed to confirm a reduction in the amount of light received due to the formation of dewdrops followed by light transmission at the external interface. Water, oil, and glass, which are available at a low cost, were selected as internal media, i.e., filling materials for the waveguide. Figure 3a–d show the optical paths inside the simulated device, with and without dew, for various internal media, i.e., air ($n_{\mathrm{IM}1}=1.000$), water ($n_{\mathrm{IM}1}=1.330$), oil ($n_{\mathrm{IM}1}=1.405$), and glass ($n_{\mathrm{IM}1}=1.468$). Under the conditions with dew, as the absolute refractive index of the filling material decreased, the density of simulated rays at the light-receiving part decreased (Figure 3a–d).

**Figure 4:**
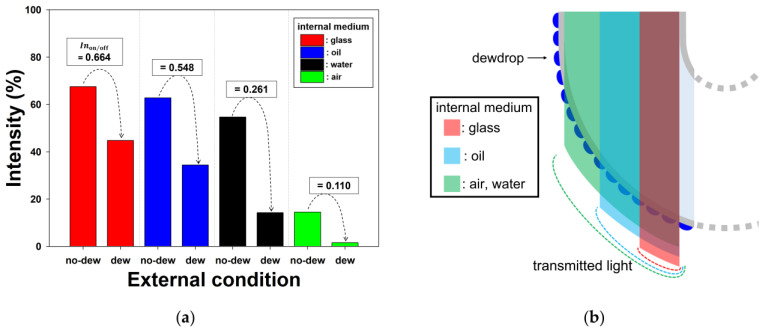
In the optical simulation results: (**a**) variations in the intensity according to the simulated dew condensation for the various internal media, and (**b**) the regions on the waveguide surface where the incident light rays are transmitted under conditions with dew for the various internal media.

Figure 4a shows the specific intensity values at the light-receiving part, with and without dew, for the various filling materials. In contrast to the non-air (i.e., water, oil, and glass) samples, the air sample exhibited a relatively low intensity under conditions without dew. The relatively low intensity in the air sample without dew was attributed to the waste of incident rays due to the transmission at Region A. The intensity on/off ratio was defined as $In_{\mathrm{on}/\mathrm{off}}=\frac{In_{\mathrm{on}}}{In_{\mathrm{off}}}$, where *In*_off_ and *In*_on_ are the intensities without dew and with dew, respectively. The *In*_on/off_ values of the air, water, oil, and glass samples were 0.11, 0.261, 0.548, and 0.664, respectively. Although the air sample exhibited a smaller *In*_on/off_ than the non-air samples in the simulation, the application of air is unlikely to realize a sufficiently clear response to dew condensation because of the relatively low specific heat.

In the non-air samples, the water sample exhibited the smallest *In*_on/off_, which was due to the largest transmission area at Region A under conditions with dew (Figure 4b). For a given curvature (i.e., fixed *R*), as the incident ray's position *x* increases along the curved interface, the incident angle *θ*_1_ decreases due to the curvature. Considering the aforementioned relationship: $\sin\;\theta_{1}=\frac{n_{\mathrm{EM}}}{n_{\mathrm{IM}1}}$ (i.e., $\theta_{1}=\sin^{-1}\left(\frac{n_{\mathrm{EM}}}{n_{\mathrm{IM}1}}\right)$) where *θ*_1_ is a specific value for $\theta_{2}=\theta_{c}$, as the filling material’s refractive index *n*_IM1_ increases, the specific *θ*_1_ for $\theta_{2}=\theta_{c}$ decreases with a consequent reduction in the transmission area at Region A. Hence, in contrast to the water sample, the glass sample with the largest *n*_IM1_ exhibited the largest *In*_on/off_ due to the smallest transmission area at Region A under conditions with dew (Figure 4b).

### 3.4. The Demonstration of Dew-Condensation Detection with the Actual Sensor

**Figure 5:**
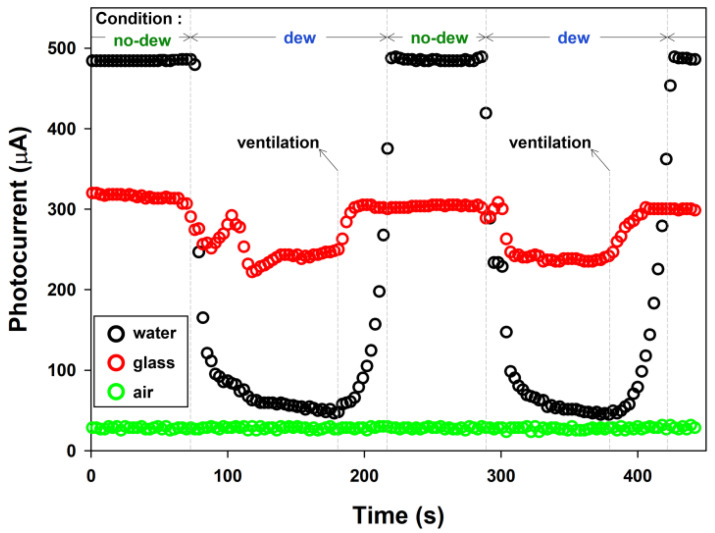
Responses of the actual sensors with the water-, glass-, and air-filled waveguides to dew condensation (the laser light wavelength: 650 nm).

Figure 5 shows variations in the photocurrent through the photodiode over time. Actual dew-condensation sensors were fabricated using air, water, and glass as internal media for the waveguide. The responses of the sensors with various media to dew condensation were compared and evaluated. The sensor with the air-filled waveguide failed to detect dew condensation as no recognizable variation in the photocurrent occurred. A sufficient number of dewdrops were not formed on the surface of the air-filled waveguide, which was attributed to the relatively low specific heat of air. Moreover, the photocurrent of the air-filled waveguide without dew was excessively low, which possibly contributed to the poor response as well. By contrast, the sensor with the water-filled waveguide successfully detected dew condensation with a clear gap between the photocurrent levels, without dew and with dew. The maximum photocurrent on/off ratio was defined as $PC_{\mathrm{on}/\mathrm{off}}=\frac{PC_{\mathrm{on}}}{PC_{\mathrm{off}}}$, where *PC*_off_ is the average photocurrent under conditions without dew and *PC*_on_ is the average minimum photocurrent under conditions with dew. The *PC*_on/off_ values of the water and glass samples were 0.09 and 0.74, respectively. The *PC*_on/off_ of the sensor with the water-filled waveguide was smaller than the other. The measured photocurrent at the light-receiving part decreased with dew formation; therefore, a smaller *PC*_on/off_ indicated a clearer difference between the internal light intensities, with and without dew. Since the specific heat of water is larger than the glass, a higher amount of dew was produced on the surface of the water-filled waveguide than on that of the glass-filled waveguide. That is, the surface of the water-filled waveguide was more dew-friendly than that of the glass-filled waveguide; hence, resulting in the larger gap between the photocurrent levels both with and without dew. In addition, the sensor with the water-filled waveguide clearly exhibited a high accuracy of detection for the formation and evaporation of dew. As result of the specific heat of water being four times higher than the air, the surface temperature of the water-filled waveguide was able to be effectively maintained at a relatively low level despite the ambient temperature rise. That is, the humid air in the box, formed by evaporating water from the heater, was warmer than the water-filled waveguide surface, which in other words was colder than the humid air. After ventilating the area, the formed dewdrops began to evaporate, which increased the measured photocurrent to its initial level. This indicates that the evaporation of dewdrops was not excessively hindered by the physical adsorption of H_2_O molecules on the waveguide surface.

**Figure 6:**
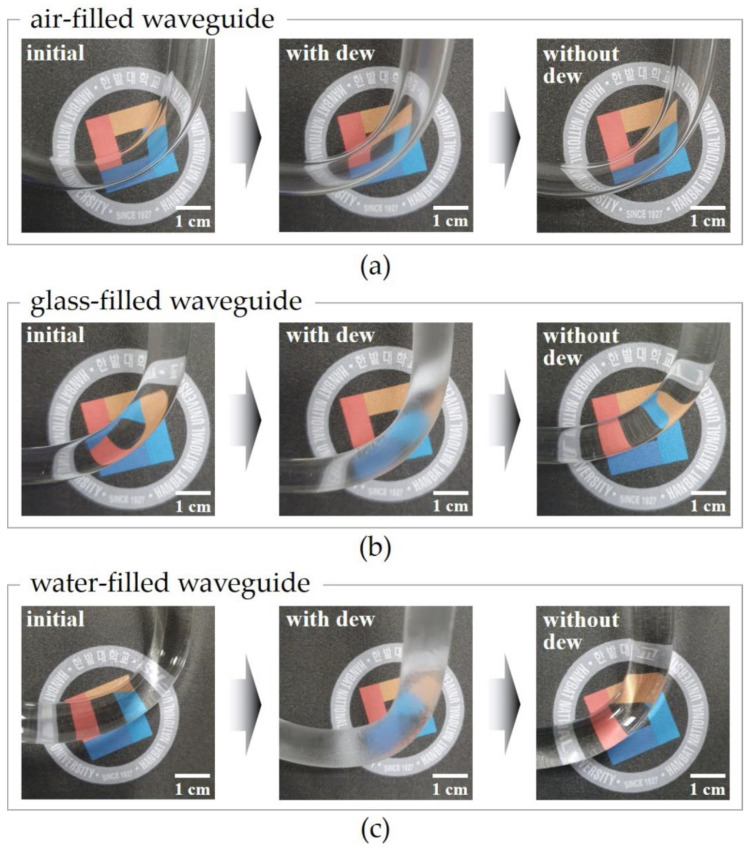
The photos of the (**a**) air-filled waveguide, (**b**) glass-filled waveguide, and (**c**) water-filled waveguide before the dew formation (‘initial’), after the dew formation (‘with dew’), and after the dew evaporation (‘without dew’) in sequence.

To confirm the dew-friendly surface of the water-filled waveguide, we took photos of the different waveguides including the water-filled waveguide before the dew formation (i.e., without dew), with dew, and without dew in sequence (Figure 6). The known specific heat values for air, pyrex glass, and water are ≈1.0, ≈0.8, and ≈4.2 J/gK, respectively [10,11,31]. The specific heat of water is distinctively higher than the others. The specific heat of the pyrex glass is comparable with that of the air. As we experience in daily life, when dewdrops are formed on a glass surface, the opaqueness of the surface increases, making it possible to recognize the presence of dew. Between the different waveguides, the water-filled waveguide exhibited the most significant variation in opaqueness under conditions with dew, which is because its surface had the highest amount of dew accumulation. The air-filled waveguide exhibited the smallest variation in opaqueness under conditions with dew. Although the specific heat of air is slightly larger than that of pyrex glass, the heat capacity of the air-filled waveguide, considering the mass as well, was obviously smaller than that of the glass-filled waveguide. Hence, the air-filled waveguide exhibited a smaller variation in opaqueness under conditions with dew than the glass-filled waveguide. After the dew evaporated, the opaqueness of all the surfaces decreased to their initial level Note that the dew condensation trends on the surfaces of the different waveguides coincided with the trends of the photocurrent variations for the actual sensors with the different waveguides.

### 3.5. A Repeatability Test of the Actual Sensor with the Water-Filled Waveguide

**Figure 7:**
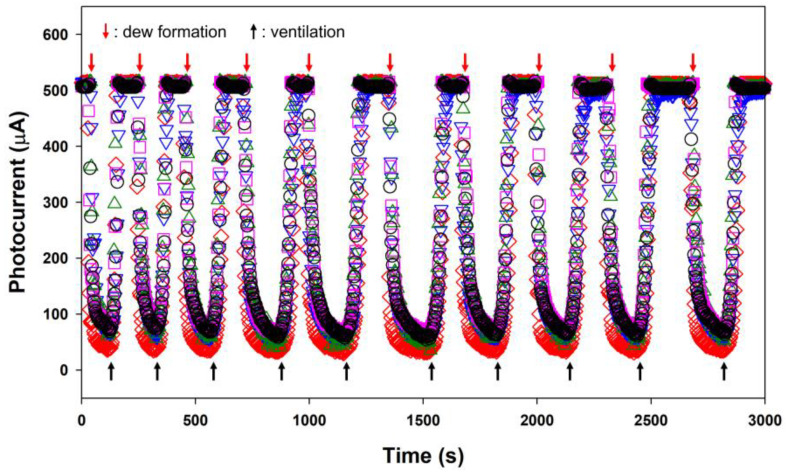
Responses of the five actual sensors with the water-filled waveguides to dew condensation.

Repeatability tests with ten consecutive measurements were performed using five fabricated sensors with water-filled waveguides. These water-filled waveguides were individual waveguides. Figure 7 shows the corresponding variations in the measured photocurrent over time. The sensor exhibited excellent repeatability characteristics, maintaining nearly constant *PC*_on_ and *PC*_off_, respectively, under the repeated formation and evaporation of the dew. In addition, the five fabricated sensors showed high uniformity as indicated by their comparable *PC*_on/off_ and repeatability characteristics. The excellent repeatability of the sensor was attributed to the chemically inert surface of the glass waveguide. The mean *PC*_off_ for the sample of five sensors was 507.9 µA with a standard deviation of 2.4 µA. The mean *PC*_on_ for the five sensors was 54.9 µA with a standard deviation of 13.1 µA. Future work needs to investigate the interactions between the waveguide surface and the dewdrops for a more thorough emulation of the trends of dew condensation on the surfaces of various crops. The surface roughness, surface treatment, and various chemical properties of waveguides will be important considerations. Polymeric plastic waveguides are a promising candidate in terms of cost reduction. In addition, if the sensor is used in different environments for different purposes, data denoising can also be considered to improve the performance of the sensor [32].

## 4. Conclusions

In summary, a low-cost dew-condensation sensor, which consists of a laser, curved water-filled waveguide, and photodiode, was developed. Dew formation and evaporation on the dew-friendly surface of the waveguide led to variations in the relative refractive index, thereby varying the measured photocurrent. In the simulations, the application of water as an internal medium for the waveguide showed a relatively small *In*_on/off_, compared to the oil and glass samples. In the actual experiments, the sensor with the water-filled waveguide exhibited a lower *PC*_on/off_ than those with the glass- and air-filled waveguides; however, *PC*_on/off_ could not be defined for the air-filled waveguides as no detection accuracy was obtained. The lower *PC*_on/off_ of the water-filled waveguide was attributed to the relatively high specific heat of water compared to that of air and glass. Lastly, the excellent repeatability and high uniformity of the sensor with the water-filled waveguide was demonstrated. These results will contribute to increasing the applicability of refractive index-based optical sensors and improving crop yield.

## Data Availability

The data presented in this study are available on request from the corresponding author. The data are not publicly available due to privacy.
